# Mucoadhesive Drug Delivery Systems for Oral Chronic Inflammatory Mucosal Diseases. The Future is Already Present. A Systematic Review

**DOI:** 10.1111/odi.70042

**Published:** 2025-07-23

**Authors:** M. E. Mauceri, M. Coppini, V. De Caro, G. Di Prima, R. Mauceri, V. Panzarella, G. Giuliana, G. Campisi

**Affiliations:** ^1^ Dipartimento di Scienze e Tecnologie Biologiche Chimiche e Farmaceutiche Università degli Studi di Palermo Palermo Italy; ^2^ Dipartimento di Medicina di Precisione in Area Medica, Chirurgica e Critica (Me.Pre.C.C.) Università degli Studi di Palermo Palermo Italy; ^3^ Dipartimento di Scienze Biomediche, Odontoiatriche e delle Immagini Morfologiche e Funzionali Università degli Studi di Messina Messina Italy; ^4^ Unità di Medicina Orale e Odontoiatria per Pazienti Fragili, Dipartimento di Riabilitazione, Fragilità e Continuità delle Cure Azienda Ospedaliera Universitaria di Palermo Palermo Italy; ^5^ Dipartimento di Biomedicina, Neuroscienze e Diagnostica avanzata (Bi.N.D.) Università degli Studi di Palermo Palermo Italy

**Keywords:** buccal disease, buccal tablet, mucoadhesive film, mucoadhesive patch, oral lichen planus, recurrent aphthous stomatitis

## Abstract

**Background:**

Oral lichen planus (OLP) and recurrent aphthous stomatitis (RAS) are considered the most painful oral inflammatory diseases, with high global prevalence and significant impacts on patients' quality of life. In the last decades, researchers have been exploring mucoadhesive drug delivery systems (MDDS) to improve drugs safety and efficacy through optimized formulations for enhancing patients compliance while addressing the limitations of semisolid products.

**Objective:**

To assess the clinical efficacy of different MDDS (e.g., tablets, films, and patches) in treating OLP or RAS by a revision of the literature.

**Methods:**

According to PRISMA guidelines, the study assessed the remission of signs and symptoms in terms of decreasing burning sensation, pain severity, and lesion size. The protocol was registered on the PROSPERO database (CRD42024603173) and records identified by Medline/PubMed/Scopus.

**Results:**

Thirteen observational studies involving humans affected by OLP or RAS were included. The absence of standardized therapies in terms of MDDS, drug, and dosing regimen was highlighted. All MDDS offered several benefits, including prolonged drug release, protection from mechanical irritation, and pain and inflammation reduction. MDDS improved patients' adherence by minimizing daily applications and systemic side effects.

**Conclusions:**

MDDS may represent a promising option for both OLP and RAS treatments, addressing key limitations of traditional formulations.

## 1 Introduction

Diseases of oral mucosa can be attributed to several conditions, ranging from immune system alterations to adverse drug events (Coppini et al. 2024; Mauceri et al. 2024; Yuan and Woo 2020).

The effects of these disorders significantly impact patients, potentially causing difficulties with speaking, eating, drinking, swallowing, and other daily activities, thereby affecting their quality of life and social interactions.

Among all the oral conditions, chronic inflammatory diseases are certainly worthy of mention especially because they require long‐term management and can be debilitating for the patient, profoundly impacting their quality of life (Rivera et al. 2022; Sanchez‐Bernal et al. 2020; Yuwanati et al. 2021).

These diseases can have an autoimmune (e.g., Pemphigus Vulgaris, Mucous Membrane Pemphigoid, etc.), infectious (e.g., Chronic Atrophic Candidiasis), or unknown, multifactorial/idiopathic immune origin (e.g., oral lichen planus (OLP), recurrent aphthous stomatitis (RAS), granulomatous cheilitis, oral granulomatosis, and oral erythema multiforme).

Among all the mentioned conditions, OLP and RAS undoubtedly have the highest global prevalence and the most significant impact on patients' quality of life (Rivera et al. 2022; Sanchez‐Bernal et al. 2020; Yuwanati et al. 2021). Given the complex and unclear etiology of both OLP and RAS, therapeutic treatments primarily aim at symptom remission, pain alleviation, and healing of lesions (Andabak‐Rogulj et al. 2023; Manfredini et al. 2021; Saeed et al. 2022).

Regarding the management of OLP and RAS, while a range of conventional pharmaceutical formulations is commercially available (e.g., mouthwashes, rinses, lozenges, tablets, ointments, suspensions, creams, and gels), their effectiveness is often hindered by limitations inherent to the oral environment, regardless of the active molecule administered (Yuan et al. 2022).

The limitations include mechanical stress, involuntary swallowing, continuous saliva flow, uneven drug distribution, and enzymatic degradation, which collectively reduce drug retention at the target site and necessitate frequent reapplications (Gupta et al. 2017; Louisy et al. 2024; Serafini et al. 2023; Tekin et al. 2024).

For this reason, over the past two decades, advancements in drug delivery technologies have sought to address these challenges through the development of mucoadhesive drug delivery systems (MDDS), including patches, tablets, film, semisolids/liquids, and particulates (Singh et al. 2023).

These innovative systems offer substantial benefits, including protection of therapeutic agents from enzymatic and systemic clearance, enhanced bioavailability and cellular uptake, and the ability to deliver immediate or controlled drug release upon administration (Kumar et al. 2022).

In designing local drug delivery devices, the selection of materials and excipients has usually been critical, as it influences key factors such as drug release kinetics, residence time in the oral cavity, and patient acceptability, including taste and texture (Kumar et al. 2022; Setty 2012; Shipp et al. 2022).

The present systematic review aims to evaluate the current evidence on the efficacy of mucoadhesive formulations in the management of chronic oral mucosal diseases, with a specific focus on their effectiveness in reducing lesion severity and symptoms of OLP and RAS.

## 2 Materials and Methods

### 2.1 Protocol

A systematic literature search was conducted independently by two authors (MC and MEM). Publications were selected and data were analyzed according to the general principles of the Cochrane Handbook for Systematic Reviews of Intervention and reported according to the Preferred Reporting Items for Systematic Review and Meta‐Analyses (PRISMA) guidelines (Page et al. 2021). The protocol was designed a priori and registered on the online database PROSPERO (CRD42024603173).

### 2.2 PICO and Research Question

The research question was designed based on PICO items in which:

- Population: Patients affected by chronic inflammatory oral mucosal disease;
- Intervention: Use of MDDS;
- Comparator: Conventional treatment or placebo;
- Outcome: Improvement of clinical signs and symptoms.

The systematic review was based on the following research question: “Is the use of MDDS effective in improving signs and symptoms in patients affected by chronic oral mucosal diseases?”

### 2.3 Data Sources and Search Strategy

A selection of studies concerning the use of MDDS in patients affected by chronic oral conditions. Records were identified using the following search engines, Medline/PubMed and *Scopus*, and by scanning reference lists of articles.

For the search strategy, MeSH terms and free text words were combined through Boolean operators as follows: (“oral ulcers” OR “Mouth Diseases” OR “Aphthous Stomatitis” OR “oral lichen planus” OR “OLP” OR “oral lesion” OR “oral disease”) AND (patches OR “mucoadhesive films” OR “drug delivery” OR “mucoadhesive drug delivery system” OR “Nanoparticle”).

The research was completed in August 2024.

### 2.4 Eligibility Criteria

The inclusion criteria for the studies are as follows:

- Human studies;
- English Language;
- Use of MDDS (e.g., patches, film, tablets);
- Patients with chronic/recurrent inflammatory conditions of the oral cavity (e.g., OLP, RAS);
- Analysis of the results in terms of reducing pain and burning sensation, as well as their effects on the improvement of oral lesions.

Exclusion criteria were studies using delivery systems other than patches, film, or tablets; narrative and systematic reviews and meta‐analyses; in vitro studies; and animal studies. Furthermore, studies analyzing semisolid dosage forms (e.g., gels and ointments) were excluded due to the uncertainty of the drug dose administered resulting from the application method, saliva washout, and potential swallowing; this is despite the use of mucoadhesive formulations that improve adhesion to the mucosa and prolong drug contact time at the application site (De Caro et al. 2021).

### 2.5 Studies Selection and Data Collection Process

The selection process was performed in two rounds. In the first round, two independent authors (MC and MEM) screened the studies reading the title and abstract, while in the second phase, a full‐text evaluation was performed. In case of disagreement between the reviewers, a final decision for the inclusion was taken in a joint session with a third author (R.M.).

**FIGURE 1:**
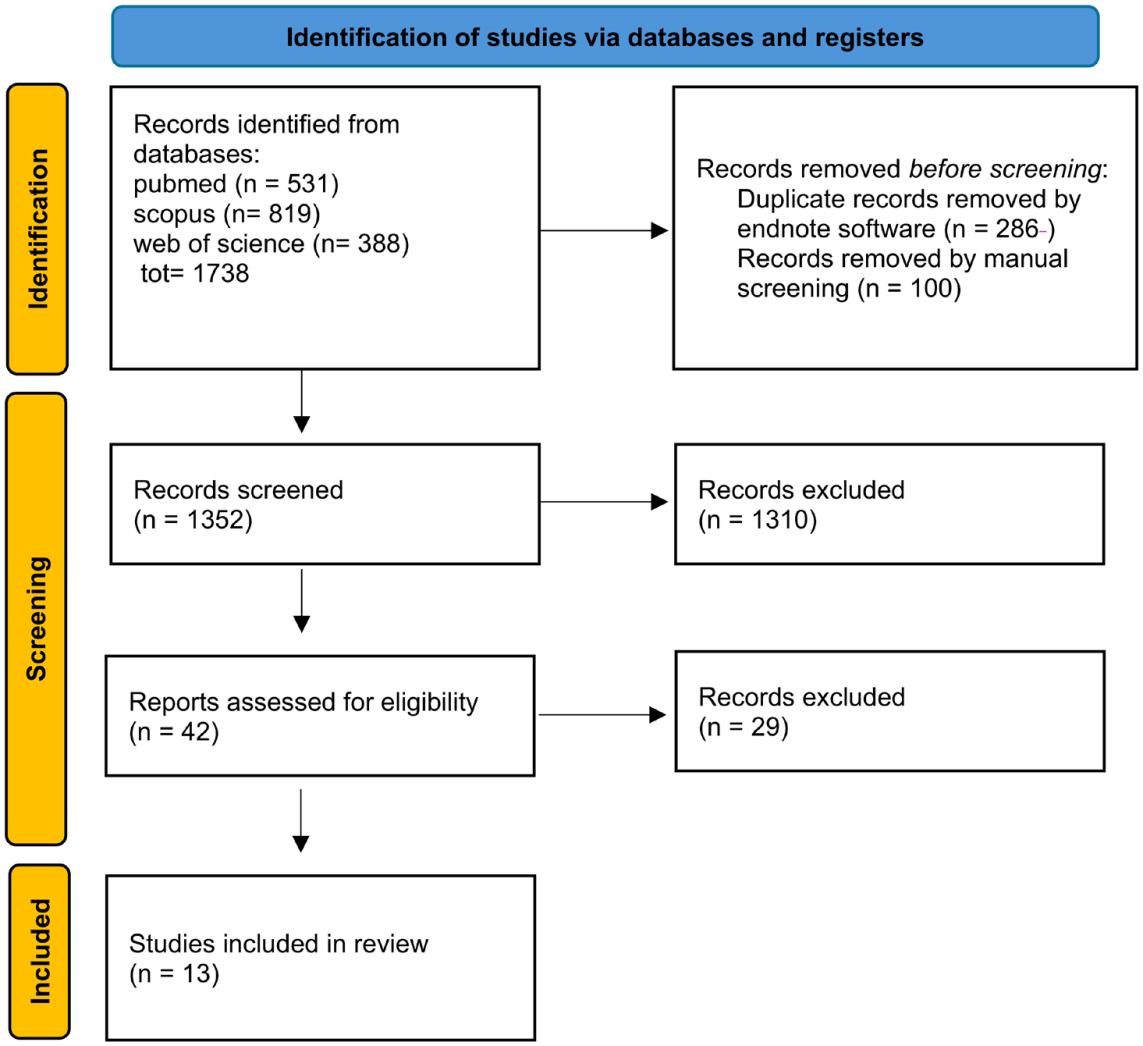
PRISMA flowchart.

The initial search strategy identified 1738 records, of which studies were excluded based on the inclusion criteria A total of 386 studies were excluded as they were identified as duplicates. The screening process of 1352 studies was performed based on the title and abstract, and 1310 records were excluded. Subsequently, a full‐text evaluation of 42 studies was carried out. Finally, 13 records were excluded, and papers were included in the current review; a detailed flow chart of the selection process is provided in Figure 1.

### 2.6 Risk of Bias Assessment

The analysis of the risk of bias in the studies included was performed according to the Cochrane Risk of Bias in randomized interventional studies tool (RoB 2) in the last version, dated 22 August 2019 (Sterne et al. 2019). The evaluation was specific to estimate the relative effect of two interventions on a target outcome. The study participants received various treatments involving different active ingredients and delivery methods (mucoadhesive patches, films, and tablets) in the intervention group, while the control group received placebo or other conventional therapies. The primary objective of all studies was to assess the effectiveness in reducing signs and symptoms. Quality assessment was performed independently by two authors (M.E.M. and M.C.), and any disagreements were resolved in a joint meeting with a third reviewer (R.M.). To assess the risk of bias in the included RCTs, five aspects were evaluated, as follows:

1. Randomization process
2. Deviation from intervention
3. Missing outcome data
4. Measurement of the outcome
5. Selection of reported result

The assessment results were scored as high, some concerns, and low risk.

### 2.7 Statistical Analysis

Firstly, a review of the selected studies was conducted to identify the outcomes of interest. Given the substantial heterogeneity among the included studies, particularly in terms of the active ingredients used in both case and control groups, delivery systems, therapeutic regimens, and outcome measurement scales, a qualitative approach to data synthesis was deemed the most appropriate. For each study, relevant data were extracted using a pre‐designed data extraction Excel sheet. The following parameters were collected:

- Study characteristics: Name of the first author, year of publication, name of the country where the study was performed, study design, and the specific pathology affecting the study population.
- Case‐control group characteristics: Description of the case population, age and sex distribution of the case population, description of the control population, and age and sex distribution of the control population.
- Methodology: Duration and administration protocol of therapy using MDDS (e.g., patch, film, or tablet).
- Obtained results: Differences in outcomes between Case and Control Groups. Subsequent analysis of the extracted data was performed using a descriptive approach.

## 3 Results

### 3.1 Characteristics of Trials

Thirteen articles published between 2004 and 2024 were included in this review. The main characteristics of the selected studies, including the type of drug delivery, active ingredient used, and outcomes, are summarized in Table 1.

**TABLE 1:** Summarization of the main characteristics and outcomes of the studies included.

No	Oral disease	*N* total patients	Gender (F/M)	Age	*N* patients test group	Intervention test group	*N* patients control group	Control group	Duration therapy	Outcomes	References
1	OLP	20	13/7	53.4 ± 12.2	10	Mucoadhesive patch containing *N. sativa* 10%	10	Mucoadhesive patches containing Triamcinolone acetonide 0.1%	3 times/day for 4 weeks	No statistically significant differences between intervention and control groups	Pakfetrat et al. (2024)
2	RAS	47	26/21	29.70 ± 6.10	23	Caffeic acid mucoadhesive tablet	24	Placebo	3 times/day for 7 days	Better results in the intervention group	Salehi et al. (2024)
3	OLP	30	Test group: 10 F Control group 1: 8/2 Control group 2: 9/1	Test group: 55.8 Control group 1: 46.6 Control group 2: 41.1	10	Tacrolimus buccal mucoadhesive patch	20	Control group 1: Tacrolimus gel Control group 2: Triamcinolone acetonide gel	Test group: 2 patch/day for 8 weeks Control group 1: 4 times/day for 8 weeks Control group 2: 4 times/day for 8 weeks	Both Tacrolimus patch and gel significantly decreased pain and lesion size than triamcinolone acetonide gel	Ibrahim et al. (2023)
4	RAS	44	36/8	Test group: 30.6 ± 6.1 Control group: 30.4 ± 5.4	22	Cinnamaldehyde mucoadhesive patch	22	Placebo	3 patch/day for 7 days	Better results in the intervention group	Molania et al. (2022)
5	OLP	122	99/39	n.d.	33 (20 μg) 34 (5 μg) 40 (1 μg)	1 or 5 or 20 μg mucoadhesive clobetasol patch (rivelin‐CLO)	31	Placebo	2 patch/day for 4 weeks	Better results in the intervention group	Brennan et al. (2022)
6	OLP	23	6/17	45.6	10 (bilateral lesions); 17 (unilateral lesion)	Mucoadhesive mycophenolate mofetil patch 2%	10	Placebo	2 patch/day for 4 weeks	Better results in the intervention group	Samiee et al. (2020)
7	RAS	24	Test group: 8/4 Control group: 8/4	Test group: 23.8 ± 1.7 (M), 22.9 ± 3.9 (F) Control group: 22.8 ± 3.3 (M), 24.6 ± 4.7 (F)	12	Mucoadhesive film of propolis extract entrapped in niosomes	12	Placebo	2 film/day	Better results in the intervention group	Arafa et al. (2018)
8	OLP	48	26/22	32–72	16	Mucoadhesive tablet containing 24 μg clobetasol‐17 propionate	Control group 1: 16 Control group 2: 16	Control group 1: commercial clobetasol ointment mixed with Orabase (123 μg/application) Control group 2: Placebo	3 tablet/day for 4 weeks	Mucoadhesive tablet appeared to be effective, avoiding the side effects of the conventional treatment	Cilurzo et al. (2010)
9	RAS	15	5/10	22–35 (26.3 ± 4.3)	n.d.	Bioadhesive patch containing licorice extract	n.d.	– Bioadhesive patches without licorice – no tratment	4 patch/day for 5 days	Similar positive results in the intervention and control groups with empty patch	Moghadamnia et al. (2009)
10	RAS	46	n.d.	n.d.	23	Oral patch containing glycyrrhiza (licorice) herbal	23	Placebo	Use patch 16 h per day for 10 days	Better results in the intervention group	Martin et al. (2008)
11	RAS	48	Test group: 18/8 Control group: 14/8	40.5–13.6 (47.8 ± 14.7)	26	Mucoadhesive patch containing citrus oil and magnesium salt	22	Oral solution containing benzocaine and benzoin tincture	3 patch/day	Mucoadhesive patch was found to be significantly more effective	Shemer et al. (2008)
12	RAS	248	n.d.	10–75	n.d.	Mucoadhesive tablet loaded with citrus oil and magnesium salt	n.d.	Placebo	n.d.	Better results in the intervention group	Mizrahi et al. (2004)
13	RAS	15	5/10	22.9 ± 2.2	n.d.	Mucoadhesive patch containing ginger officinale extract	n.d.	Placebo	4 patch/day: for 20 min after every meal and before going to bed for 7 days	No statistically significant differences between intervention and control groups	Haghpanah et al. (2015)

Six studies were conducted in Iran (Haghpanah et al. 2015; Moghadamnia et al. 2009; Molania et al. 2022; Pakfetrat et al. 2024; Salehi et al. 2024; Samiee et al. 2020), two in Egypt (Arafa et al. 2018; Ibrahim et al. 2023), two in the USA (Brennan et al. 2022; Martin et al. 2008), two in Israel (Mizrahi et al. 2004; Shemer et al. 2008), and one in Italy (Cilurzo et al. 2010). Among these, all were randomized clinical trials, except one which was non‐randomized (Pakfetrat et al. 2024).

Studies included 730 patients, of whom 460 with gender definition (291 female and 169 male), while two studies did not report gender distribution (Martin et al. 2008; Mizrahi et al. 2004).

The participants' ages ranged from 10 to 75 years, but it was not possible to calculate an average age since most studies provided only aggregated data, as most studies did not specify the participants' ages.

The studies focused on two main chronic oral conditions: OLP and RAS. OLP diagnosis has been performed by incorporating both clinical and histopathological features (Idrees et al. 2021).

Among the participants, 243 were diagnosed with OLP (Brennan et al. 2022; Cilurzo et al. 2010; Ibrahim et al. 2023; Pakfetrat et al. 2024; Samiee et al. 2020) and 239 with RAS (Arafa et al. 2018; Haghpanah et al. 2015; Martin et al. 2008; Mizrahi et al. 2004; Moghadamnia et al. 2009; Molania et al. 2022; Salehi et al. 2024; Shemer et al. 2008).

One study differentiated between RAS (154 patients) and single aphthous ulcers (94 patients) (Mizrahi et al. 2004).

Mucoadhesive tablets were used in a single study on patients with OLP (Cilurzo et al. 2010) and in two studies on patients with RAS (Mizrahi et al. 2004; Salehi et al. 2024).

Although in several papers the authors referred to their formulations as “patches”, they did not have an impermeable backing layer that determined a unidirectional release of the drug (as is generally stated in the definition of “patch”), rather they were thin mucoadhesive formulations that dissolved over time, making them like films. This aspect has been explored in the discussion section. For this reason, the studies have been aggregated when the so‐called film or patch referred to similar formulations that dissolved entirely. Therefore, mucoadhesive patches/films were used in four studies involving patients with OLP (Brennan et al. 2022; Ibrahim et al. 2023; Pakfetrat et al. 2024; Samiee et al. 2020) and in six studies focusing on patients with RAS (Arafa et al. 2018; Haghpanah et al. 2015; Martin et al. 2008; Moghadamnia et al. 2009; Molania et al. 2022; Shemer et al. 2008). Only one study involved the OLP treatment with a patch formulation combining a mucoadhesive porous layer containing clobetasol and an impermeable non‐adhesive backing layer (Brennan et al. 2022).

Several active ingredients were tested across the included studies. Among those focusing on patients with OLP, clobetasol 17‐propionate was employed in two studies (Brennan et al. 2022; Cilurzo et al. 2010), tacrolimus in one study (Ibrahim et al. 2023) as well as 10% *Nigella sativa* extract (Pakfetrat et al. 2024) and mycophenolate mofetil (Samiee et al. 2020).

Regarding clinical trials involving patients with RAS, several studies assessed the effectiveness of natural active ingredients, including citrus oil and magnesium salt, licorice extract (Martin et al. 2008; Mizrahi et al. 2004; Moghadamnia et al. 2009; Shemer et al. 2008), caffeic acid (Salehi et al. 2024), cinnamaldehyde (Molania et al. 2022), propolis extract encapsulated in niosomes (Arafa et al. 2018), and ginger officinale extract (Haghpanah et al. 2015).

The treatment frequency of MDDS application varied significantly across studies, ranging from one to three daily applications depending on the type of drug and formulation employed. Also, the treatment duration was not specified for all the studies, making it difficult to compare outcomes.

To treat OLP, in two included studies, therapeutic regimens employed were similar, which involved three applications a day of patches (Pakfetrat et al. 2024) and tablets (Cilurzo et al. 2010) for 4 weeks, while the other two studies involved the application of patches/films twice a day for 4 weeks (Brennan et al. 2022; Samiee et al. 2020).

Regarding RAS treatment, only in two studies were therapeutic regimens similar, which involved three applications a day for 7 days (Molania et al. 2022; Salehi et al. 2024).

Regarding the outcomes reported in all studies analyzed, the primary objective was to evaluate the effectiveness of MDDS in alleviating symptoms and reducing lesion severity.

Most of the studies used a placebo in the control group (Arafa et al. 2018; Brennan et al. 2022; Cilurzo et al. 2010; Haghpanah et al. 2015; Martin et al. 2008; Mizrahi et al. 2004; Moghadamnia et al. 2009; Molania et al. 2022; Salehi et al. 2024; Samiee et al. 2020).

Two studies used as control group the same drug as the intervention group but delivered through a conventional pharmaceutical form (Cilurzo et al. 2010; Ibrahim et al. 2023) and in one study, the placebo was an oral solution containing benzocaine, an anesthetic (Shemer et al. 2008).

The extent of the inflammatory lesions was measured in mm or cm and sometimes using a caliper. All studies investigated pain and burning intensity using the Visual Analog Scale (VAS), a tool that allows patients to rate the intensity of pain or burning sensation on a continuous scale ranging from 0 to 10. However, VAS assessments were carried out at different time points during the therapy duration, making comparisons across studies very difficult.

Moreover, the lesion severity was evaluated by different methods, among which the Tongprasom Score was chosen for the OLP evaluation. According to the last one, a rating from 0 to 5 was assigned based on the size of the lesion and clinical characteristics (e.g., white striations, atrophy, and erosion), without considering the location of the disease (Unnikrishnan et al. 2023).

In the study of Samiee et al. regarding OLP treatment, the lesion size was measured using a sterile digital caliper, and the severity of burning sensation and pain was assessed using VAS. In this study, a significant reduction in pain, burning sensation, and lesion size was observed only after the fourth week of treatment, in particular demonstrating that the drug effects on lesion reduction were time‐dependent, whereas the empty device was effective only in VAS reduction (Samiee et al. 2020).

In the study of Moghadamnia et al. on RAS treatment, despite a reduction in VAS being observed after the application of licorice patches on days 2, 3, 4, and 5 compared to the no‐treatment group (*p* < 0.001), it was highlighted that licorice‐containing patches were almost as effective as those without licorice, suggesting that the mechanical protection of the mucosa was essential in reducing pain and promoting healing (Moghadamnia et al. 2009).

Interesting was the study of Pakfetrat et al. in which the intervention group was treated with mucoadhesive patches containing *Nigella sativa* extract 10%, while the control group was treated with mucoadhesive patches containing triamcinolone acetonide 0.1% (Pakfetrat et al. 2024).

In this study, the assessment was conducted using the VAS and Tongprasom scoring systems at five different time points. After the examination sessions, based on repeated measures analysis, the reduction in both variables demonstrated statistical significance over the 4‐week period in both the *Nigella sativa* and triamcinolone groups, yielding *p* values of < 0.001 for both variables in both groups. Although no statistically significant differences between the treatments were observed, the study demonstrated that the positive results in terms of symptom relief were obtained; thanks to the patches application further highlighting that *Nigella Sativa* was as effective as corticosteroids, with additional safety advantages. In this study, the mucoadhesive patches were prepared using a two‐layer structure (Pakfetrat et al. 2024).

By correlating the effectiveness of MDDS on treated pathologies, patients affected by OLP reported significant improvements in the test groups compared to the control groups. The active ingredients evaluated in these studies included tacrolimus administered by single‐layer patch (Ibrahim et al. 2023), clobetasol administered by tablet and bilayer patch (Brennan et al. 2022; Cilurzo et al. 2010) and mycophenolate mofetil administered by mucoadhesive patch (Samiee et al. 2020). For patients affected by RAS, notable improvements in the test groups compared to the control group were reported. In these studies, the MDDS employed were mucoadhesive tablets containing caffeic acid (Salehi et al. 2024) or a combination of citrus oil and magnesium salt (Mizrahi et al. 2004; Shemer et al. 2008) or cinnamaldehyde (Molania et al. 2022), films containing propolis extract (Arafa et al. 2018), and patches loaded with licorice extract.

(Martin et al. 2008).

In the remaining three studies, when the MDDS employed were licorice extract loaded patch (Moghadamnia et al. 2009) and ginger officinale extract loaded film (Haghpanah et al. 2015) no statistically significant signs and symptoms improvement was observed. The main characteristics of the studies included were summarized in Table 1.

### 3.2 Risk of Bias

Based on the RoB2 assessment for randomized trials, most studies were rated as having a high risk of bias due to the use of non‐specific outcome measurement methods (Arafa et al. 2018; Mizrahi et al. 2004; Cilurzo et al. 2010; Shemer et al. 2008), while one study was assessed as having a high risk of bias because it was a non‐randomized pilot study (Pakfetrat et al. 2024). Three studies were judged to be of some concern, primarily due to unspecified randomization methods or unclear blinding procedures, specifically regarding whether outcome assessors or intervention providers were aware of participants' assigned interventions during the trial (Moghadamnia et al. 2009; Ibrahim et al. 2023; Samiee et al. 2020).

The remaining studies were evaluated as having a low risk of bias (Salehi et al. 2024; Haghpanah et al. 2015; Molania et al. 2022; Martin et al. 2008; Brennan et al. 2022). The risk of bias assessment is reported in Table S1.

## 4 Discussion

The present systematic review aims to evaluate the effectiveness of MDDS (e.g., patches, films, tablets) used to date in treating chronic mucosal diseases of the oral cavity regarding their ability to reduce pain and lesion severity.

Prior to this systematic review, no effort had been made to organize the numerous proposed pharmaceutical technologies systematically, nor to identify the most suitable approach for each specific disease and intraoral site.

After screening, 13 studies were included in our systematic review, which focused exclusively on OLP and RAS treatments using MDDS. The studies included have confirmed, in general, the potential usefulness of mucoadhesive patches as innovative formulations in treating at least two prevalent chronic oral conditions (OLP and RAS). The main findings include therapeutic efficacy in pain reduction and a decrease in lesion severity and size in both conditions.

Regarding the oral application of mucoadhesive devices, no studies, to the best of our knowledge, have focused on chronic oral mucosal disease management. Differently, mucoadhesive patches have been explored for the treatment of herpes labialis, as demonstrated by the Aciclovir Lauriad buccal tablet, which provides high and prolonged acyclovir exposure in the oral cavity (Bieber et al. 2014).

In recent years, mucoadhesion has gained significant attention in pharmaceutical technology, especially within oral medicine, where ongoing pharmacological research has enhanced the development of MDDS, leading to more efficient and targeted drug delivery approaches (Singh et al. 2023).

Mucoadhesion, the interaction between a mucosal surface and a natural or synthetic polymer, involves a complex three‐step process. The first is the contact stage, where the mucoadhesive material comes into contact with the mucous membrane, and wetting and spreading increase the interaction surface (Subramanian 2021). This is followed by the interpenetration stage, in which the polymer chains diffuse into the mucus layer, creating a stronger bond. Lastly, during the consolidation stage, mechanical and chemical forces reinforce the adhesion, ensuring a stable and long‐lasting attachment (Shaikh et al. 2011; Smart 2005).

The oral mucoadhesive delivery system includes tablets, films, patches, semisolids/liquids, and particulates. Despite their advantages over conventional therapies, mucoadhesive formulations face several challenges that can affect their efficacy. Variations in oral mucosal structure, saliva flow, and individual factors like tongue movement can impact adhesion and performance. Additionally, the limited area available for application restricts the amount of drug that can be loaded and delivered accordingly, thus affecting the formulation's overall efficacy. Lastly, user comfort is a key factor, as poorly designed mucoadhesive formulations may lead to discomfort, irritation, or difficulties during application, which can negatively affect treatment adherence (Boddupalli et al. 2010).

Buccal tablets are small, flat, and oval dosage forms, with a diameter ranging from 5 to 8 mm, designed to adhere to the cheek and gum for local or systemic drug delivery. When applied, they soften, adhere to the mucosa, and remain in place until the dissolution or drug release is complete. Buccal tablets, which can be double or multilayered, are made using bioadhesive polymers and additives which are typically formulated through direct compression of powders (Pushadapu et al. 2025). Depending on the additives used, these tablets can deliver the drug in multiple directions, either to the mucosal surface or into the oral cavity. Though effective, their main drawbacks include poor mouth feel, taste, irritation, and limited flexibility, which can reduce patient compliance, especially with long‐term use.

Mucoadhesive films are designed to strongly adhere to the mucosal membrane, ensuring accurate dosage and enhanced drug absorption for local and systemic treatments (De Caro et al. 2022). Made from biocompatible, biodegradable polymers, they are ideal for buccal use due to their flexibility, comfort, and durability (Di Prima et al. 2020). Compared to tablets, they offer easier use and better patient compliance. FDA‐approved buccal films include those for buprenorphine, fentanyl, naloxone, and lidocaine. Advanced methods like solvent casting and 3D printing allow scalable, customizable drug release and dosing profiles (Jacob et al. 2020). Additionally, mucoadhesive films provide prolonged retention compared to oral gels, which are readily diluted or removed by saliva. In the context of local oral treatments, these films also contribute to wound protection, pain relief, and improved therapeutic effectiveness (Alves et al. 2020; De Caro et al. 2019).

Buccal patches have become increasingly popular in drug delivery due to their high patient acceptance, being easy to apply, flexible, and comfortable. Furthermore, buccal patches provide a safe and convenient delivery method, as the drug absorption can be immediately stopped by simply removing the patch if any undesirable effects occur. Generally, patches are non‐dissolving, matrix‐modified release dosage forms, typically featuring a laminated structure with a nonporous backing layer and a drug‐infused mucoadhesive layer that adheres to the oral mucosa. They release the drug either one‐ or two‐directionally into the mucosa or oral cavity. Patch designs include single‐layer, dual‐layer (with a backing to direct release), and triple‐layer systems for controlled drug delivery (Shirvan et al. 2019). Recent literature reports numerous patch formulations for both topical and systemic use, often developed and characterized using the same methods as buccal films (Mann Garima et al. 2022). Notably, over the past decade, the definition of “patch” has gradually lost the necessary presence of an impermeable layer. As a result, patches are now described that, due to their production methods (e.g., solvent casting) and composition (e.g., biodegradable polymers), are entirely comparable to films (Shirvan et al. 2019).

Our study suggests that the patch and film function as both a device and a vehicle for drug delivery, playing a key role in improving the patient's symptoms through their ability to protect lesions from the mechanical stress of tongue movements. Some of the studies considered support our theory. Brenna et al. compared the efficacy of mucoadhesive patches containing different percentages of clobetasol versus empty patches. Although the empty patch showed no effect on symptom relief, it produced a significant reduction in pain (Brennan et al. 2022). Moghadamnia et al. demonstrated that patches with and without licorice both resulted in a reduction in pain intensity and ulcer diameter, although there were no significant differences between the empty and loaded patches (Moghadamnia et al. 2009). Furthermore, the ginger‐loaded mucoadhesive patch and placebo patch studied by Haghpanah et al. showed a similar reduction in the severity of pain but not any significant effect on the lesion diameter (Haghpanah et al. 2015). These results highlight the importance of the dosage form used.

Another study compared tacrolimus in gel and patch forms against a placebo for OLP treatment. The mucoadhesive patch showed superior efficacy, providing greater symptom relief, reducing inflammation, and better controlling caspase‐3 expression. Its prolonged drug release and improved adhesion enhanced treatment effectiveness and patient compliance, outperforming the gel (Ibrahim et al. 2023).

Although the results did not reach statistical significance, promising findings were noted concerning the effectiveness of patches in treating RAS. Notably, all studies employed natural active compounds, with none utilizing first‐line treatments such as clobetasol.

Based on the analyzed data, MDDS are emerging as a novel and promising therapeutic strategy for the treatment of OLP and RAS. These systems have consistently shown effectiveness in decreasing lesion size, relieving pain, and promoting mucosal healing. Their capacity to deliver active agents directly to the lesion site through a sustained release mechanism enhances therapeutic outcomes while reducing systemic adverse effects. This approach makes MDDS a more patient‐friendly option, as demonstrated by higher compliance rates, attributed to the reduced frequency of application compared to traditional topical therapies.

Additionally, it is well‐established that first‐line treatments for OLP and RAS typically involve long‐term topical corticosteroid use, which can cause side effects such as dysgeusia, xerostomia, nausea, and a decrease in local immune defenses, leading to superinfections like candidiasis. A key advantage of MDDS is their ability to reduce these side effects, thereby enhancing patient compliance.

The safety profile of MDDS further strengthens their potential as a treatment option. Reported adverse effects are minimal, with only mild bitterness occasionally noted, underscoring their tolerability and acceptability among patients. Their versatility is exemplified by the diverse range of active agents incorporated into these devices. Synthetic compounds, including clobetasol, tacrolimus, and mycophenolate mofetil, have shown robust anti‐inflammatory and immunosuppressive effects, significantly mitigating the severity and extent of lesions. Simultaneously, natural agents such as *Nigella sativa* , propolis, citrus oil, and magnesium salts have demonstrated comparable efficacy, leveraging their anti‐inflammatory, antimicrobial, and wound‐healing properties while maintaining a low side‐effect profile.

Despite these promising findings, the literature highlights significant gaps that warrant further investigation. Unresolved questions regarding optimal dose regimen, treatment duration, and long‐term safety require systematic exploration. Additionally, the absence of standardized formulations and protocols poses challenges to ensuring consistent clinical outcomes. The significant heterogeneity in the reported data—both in terms of composition and methodology—reflects the lack of consolidated standardization in the pharmaceutical dosage forms. Such variability not only complicates direct comparisons between studies but also impacts the reproducibility of results, which is crucial for translating findings into clinical practice.

The present study possesses some limitations including the heterogeneity of included studies in terms of oral diseases, active ingredients employed, and the use of different MDDS; this made it difficult to compare the results observed. Moreover, not all studies have assessed oral diseases in the same way and with the same time intervals, and few studies analyzed MDDS therapy compared to conventional therapy of oral chronic diseases, limiting the generalizability of the results (Cilurzo et al. 2010; Ibrahim et al. 2023).

Therefore, increased scientific rigor is required in the future development of clinical protocols for the assessment of MDDS efficacy. Specifically, studies should systematically evaluate:

– The intrinsic efficacy of active ingredients, particularly those of natural origin, through comparative clinical trials between formulations containing the active drug and placebo formulations and
– The efficacy of MDDS, via comparative clinical trials between MDDS and conventional formulations (such as gels, ointments, pastes), both containing the active drug (already established as effective).

Furthermore, these studies should employ symptoms and outcomes assessment scales that are recognized and widely adopted by the international scientific community (e.g., VAS, wound measurement, Tongprasom Score, etc.) and by selecting a reasonable time interval for collecting outcomes (e.g., weekly).

Future clinical trials exploring the use of MDDS in oral lesions are needed to fully harness the therapeutic potential of these innovative drug delivery systems to facilitate their widespread adoption in clinical practice, ultimately benefiting patients who suffer from these chronic oral conditions.

## 5 Conclusions

Although the current literature provides limited systematic guidance on selecting the most suitable devices and formulations for specific clinical applications, and the risk of bias assessment reports a high number of studies with some concerns regarding randomization and blinding procedures, our findings indicate that MDDS (such as patches, tablets, and films) used in the treatment of RAS and OLP represent a promising approach to enhance the efficacy of active ingredients by optimizing their release profile, absorption, and distribution. The authors suggest that if future studies rigorously confirm the efficacy of drugs delivered through MDDS, these devices could become the new gold standard for treating various oral conditions. This study emphasizes that MDDS enhance therapeutic outcomes by reducing the frequency of applications, thereby improving patient compliance and adherence to treatment. Finally, the properties of MDDS demonstrate a favorable safety profile with few or no side effects, making them especially suitable for long‐term use in chronic conditions.

## Author Contributions

**M. E. Mauceri:** methodology, formal analysis, investigation, writing – original draft. **M. Coppini:** methodology, formal analysis, validation, writing – original draft. **V. De Caro:** conceptualization, writing – review and editing, supervision, funding acquisition. **G. Di Prima:** writing – review and editing, formal analysis, methodology. **R. Mauceri:** validation, investigation, writing – review and editing, formal analysis. **V. Panzarella:** validation, investigation. **G. Giuliana:** conceptualization, writing – review and editing, supervision. **G. Campisi:** conceptualization, funding acquisition, writing – review and editing, supervision.

## Consent

The authors have nothing to report.

## Conflicts of Interest

The authors declare no conflicts of interest.

## Supporting information


Table S1

## Data Availability

All data generated or analyzed during this study are included in this published article.
