# Supplementary material for: Mucoadhesive Drug Delivery Systems for Oral Chronic Inflammatory Mucosal Diseases. The Future is Already Present. A Systematic Review
Source: Oral Dis. 2025 Jul 23;32(1):25–36. doi: 10.1111/odi.70042 (PMC13031419; doi:10.1111/odi.70042)
Supplement: Supplementary file 1 — Table S1 [file ODI-32-25-s001.docx]

| **Supplementary Table 1. Risk of bias assesment.** | | | | | | |
| --- | --- | --- | --- | --- | --- | --- |
| **Study** | **Randomization process** | **Deviation from intervention** | **Missing outcome data** | **Measurement of the outcome** | **Selection of reported result** | **Overall rating** |
| Pakfetrat et al., 2024 | High | Some concerns | Low | High | Low | High |
| Salehi et al., 2024 | Low | Low | Low | Low | Low | Low |
| Ibrahim et al., 2023 | Low | Some concerns | Low | Low | Low | Some concerns |
| Molania et al., 2022 | Low | Low | Low | Low | Low | Low |
| Brennan et al., 2022 | Low | Low | Low | Low | Low | Low |
| Samiee et al., 2020 | Low | Some concerns | Low | Low | Low | Some concerns |
| Arafa et al., 2018 | Low | Some concerns | Low | Some concerns | Low | High |
| Cilurzo et al., 2010 | Low | Low | Low | High | Low | High |
| Moghadamnia et al., 2009 | Low | Some concerns | Low | Low | Low | Some concerns |
| Martin et al., 2008 | Low | Low | Low | Low | Low | Low |
| Shemer et al., 2008 | Low | Low | Low | High | Low | High |
| Mizrahi et al., 2004 | Low | Low | Low | High | High | High |
| Haghpanah et al., 2015 | Low | Low | Low | Low | Low | Low |
